# Dynamic coordination of the perirhinal cortical neurons supports coherent representations between task epochs

**DOI:** 10.1038/s42003-020-01129-3

**Published:** 2020-07-30

**Authors:** Tomoya Ohnuki, Yuma Osako, Hiroyuki Manabe, Yoshio Sakurai, Junya Hirokawa

**Affiliations:** 1 Laboratory of Neural Information, Graduate School of Brain Science, Doshisha University, Kyotanabe-Shi, Kyoto 610-0394 Japan; 2 Research Fellow of the Japan Society for the Promotion of Science (JSPS), Chiyoda-Ku, Tokyo 102-0083 Japan

**Keywords:** Neural encoding, Neural decoding, Decision, Cortex, Reward

## Abstract

Cortical neurons show distinct firing patterns across multiple task epochs characterized by different computations. Recent studies suggest that such distinct patterns underlie dynamic population code achieving computational flexibility, whereas neurons in some cortical areas often show coherent firing patterns across epochs. To understand how coherent single-neuron code contributes to dynamic population code, we analyzed neural responses in the rat perirhinal cortex (PRC) during cue and reward epochs of a two-alternative forced-choice task. We found that the PRC neurons often encoded the opposite choice directions between those epochs. By using principal component analysis as a population-level analysis, we identified neural subspaces associated with each epoch, which reflected coordination across the neurons. The cue and reward epochs shared neural dimensions where the choice directions were consistently discriminated. Interestingly, those dimensions were supported by dynamically changing contributions of the individual neurons. These results demonstrated heterogeneity of coherent single-neuron representations in their contributions to population code.

## Abstract (web summary)

Tomoya Ohnuki et al. show that individual neurons in the rat perirhinal cortex coherently encoded choice directions across cue and reward epochs. Individual neurons also displayed dynamically changing contributions to population encodings between these epochs, suggesting that they flexibly coordinate to support computations associated with different epochs while holding temporally coherent representations.

## Introduction

Individual neurons across cortical areas show temporally flexible responses to multiple task epochs characterized by different computational aspects, such as cue, action, and reward^1–3^. Recent studies have indicated that such diverse single-neuron responses are only interpretable in terms of their contributions to population dynamics which flexibly realize different computations, particularly in association and motor cortices^4–9^. These studies highlight the complex changes occurring in neural responses across epochs of a given task, which can provide orthogonal neural subspaces for independent computations. In contrast, individual neurons in many cortical areas have been shown to often encode relevant information across multiple epochs (anterior cingulate cortex^10^, insular cortex^11^, motor cortex^12^, orbitofrontal cortex^13–15^, and perirhinal cortex (PRC)^16^), suggesting their ability to support coherent representations through different task epochs. Because of the differences in the forms of explanations in these studies (that is, population or single-neuron level), how coherent representations carried by individual neurons can be reconciled with dynamically changing population structure has not been well investigated.

In the present study, we explored the neural responses in the PRC, which has been implicated in associative memory^17–22^. This region receives sensory inputs from almost all the modalities, reward-related signals from the amygdala, and contextual information from the prefrontal cortex, entorhinal cortex, and hippocampus^23–25^. Recent studies have shown that the PRC neurons modulate their sustained responses to visual cues as a function of time contexts^26,27^. It has also been shown that individual neurons in the PRC flexibly encode graded visual stimuli during active cue-sampling and response categories during movements for choice^17^. These results suggest the capacity of the PRC neural population to employ both population dynamics and coherent representations through multiple task epochs.

To investigate how the PRC shows coherent single-neuron code and dynamic population code across different epochs, we employed a standard two-alternative forced-choice task and analyzed neural responses in two epochs, where different computations are demanded: making predictions about the outcome of choices (cue epoch) and reinforcing the choices (reward epoch). By taking advantage of the interleaved visual and olfactory cue stimuli, which allowed us to evaluate modality-independent encodings, we analyzed dynamic population encodings related to different choices during those epochs in relation to single-neuron level selectivity. Our results suggest that individual neurons flexibly coordinate to support computations associated with different epochs, while they are holding temporally coherent representations.

## Results

### Individual neurons in the PRC encode choice directions

**Fig. 1:**
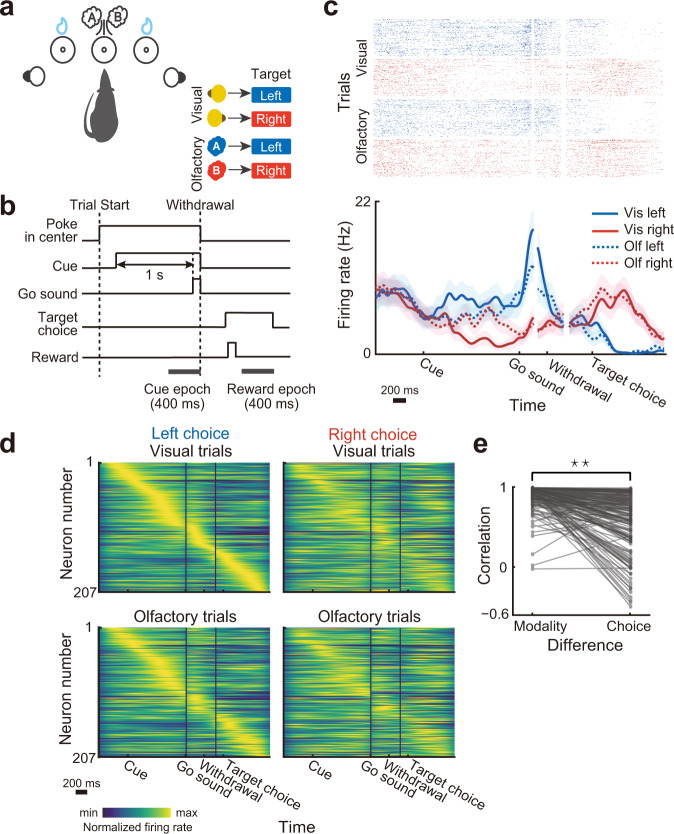
Firing patterns of the PRC neurons in a two-alternative forced-choice task. **a** Schematic drawing of the behavioral apparatus and the cue–target associative relationships in the two-alternative forced-choice task. **b** Schematic of the task timeline. **c** Raster plot and peri-event time histogram showing the response of a representative neuron. Trial types are classified according to the cue modality and target choice as follows: blue, left target choices; red, right target choices; solid line, visual trials; dashed line, olfactory trials. Neural responses in the correct trials were independently aligned to the cue, withdrawal, and target-choice onset and then reconstructed because of variable time between them. Lines and shaded areas indicate mean and s.e.m., respectively. **d** Firing patterns across all the neurons (*n* = 207) for the different trial conditions. In each trial type, the mean firing rate of each neuron was normalized to its peak. For all the trial types, the neurons were sorted by their peak firing time in the visually cued left choice trials (upper left). **e** Comparison of temporal firing patterns of individual neurons between the different cue modalities and choice directions. For comparison between the different modalities, for each neuron, a correlation coefficient was computed between peak-normalized firing rates in the visual left (upper left in **d**) and olfactory left trials (bottom left in **d**). For comparison between the different choices, a correlation coefficient was computed between peak-normalized firing rates in the visual left (upper left in **d**) and visual right trials (upper right in **d**). Nearly identical results were achieved when correlation coefficients were computed between the visual right and olfactory right trials and between the olfactory left and olfactory right trials (two-sided Wilcoxon signed-rank test; mean correlation for different modalities = 0.872 ± 0.13, mean correlation for different choice directions = 0.62 ± 0.358, *P* = 1.515 × 10^−23^).

We trained rats to perform a two-alternative forced-choice task where they chose a target port (left/right) associated with a presented cue to obtain the reward (Fig. 1a–b). The task performance was of a similar level regardless of the cue modality (mean correct rate in visual trials =  95.6 ± 5.5%; olfactory trials = 92.3 ± 4.3%). We recorded spiking activities from the left PRC (*n* = 207 neurons) during the task performance (37 sessions in five rats).

As shown in Fig. 1c, the PRC neurons typically showed distinct temporal firing patterns between the left and right trials. To characterize how the PRC was activated by different trial conditions, we compared firing patterns among the different cue modalities and choices across all the recorded neurons. The neurons were sorted by their peak firing rates in visually cued left choice trials (top left in Fig. 1d). As consistent with previous studies in other brain regions^28–33^, the peak responses of the PRC neurons tiled the duration of a trial. The response patterns across the neurons were well preserved between the cue modalities (comparison between the top and bottom in Fig. 1d) but much less so between the choice directions (comparison between the left and right in Fig. 1d). We found that the majority of the neurons showed more strongly correlated response patterns between the different modalities than between the different choice directions (two-sided Wilcoxon signed-rank test; *P* = 1.186 × 10^−24^; Fig. 1e). These results suggest that the firings of the PRC neurons are more sensitive to the choice behavior than the cue information.

**Fig. 2:**
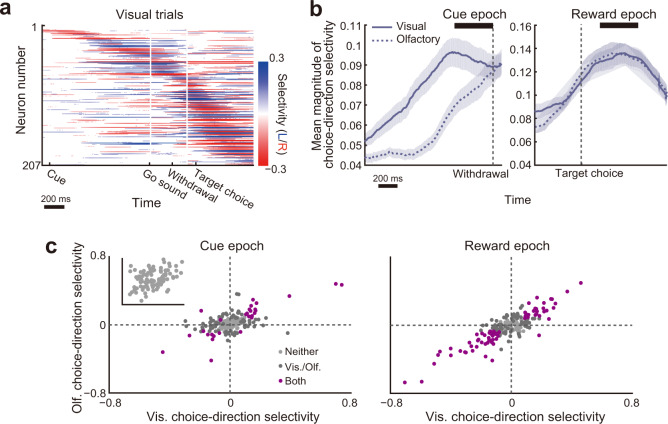
Choice-direction selectivity of the individual PRC neurons in different epochs of the task. **a** Temporal patterns of choice-direction selective responses of the PRC neurons (*n* = 207) in the correct visual trials. Colors indicate selectivity to the left (blue) or right (red) target choice. Neurons were sorted according to the time of their peak selectivity. Only segments with significant selectivity are shown (*P* < 0.05; 1000 permutations). **b** The time course of the choice-direction selectivity magnitude in the visual (solid line) and olfactory (dashed line) trials averaged across all the neurons. Neural responses around the withdrawal onset (left) and target-choice onset (right) are shown. Lines and shaded areas indicate mean and s.e.m., respectively. **c** Scatter plots showing the choice-direction selectivity of the neural population during the cue (left) and reward (right) epochs (*n* = 207 neurons). Each point corresponds to the values of a single neuron. Colors indicate significance (*P* < 0.05; 1000 permutations): light gray, no selectivity; deep gray, significant in either cue modality; purple significant in both cue modalities. Inset, the choice-direction selectivity of the non-selective neurons (*n* = 101) in the cue epoch.

We thus quantified selective responses of the individual neurons to the different choice directions by using ROC analysis. We defined “choice-direction selectivity” broadly as signals reflecting a chosen direction in each trial. The individual neurons encoded the choice directions at different time points of the trial duration (Fig. 2a for visual trials; Supplementary Fig. 1 for olfactory trials). Also, each neuron often showed such encodings at the time points other than its peak selectivity, suggesting that they flexibly respond to multiple epochs of the task. We found two epochs where the selectivity in both modalities reaches their peaks (Fig. 2b), the cue epoch (−400 to 0 ms before withdrawal from the central port) and the reward epoch (200–600 ms after target choice). Many neurons encoded the choice direction during these epochs (Fig. 2c; 51.21% and 70.53% of the neurons showed significant selectivity in the cue and reward epochs, respectively). We observed a slight bias toward the ipsilateral choice in the cue epoch (two-sided sign test; mean visual choice-direction selectivity = 0.006 ± 0.124, *P* = 0.889; mean olfactory choice-direction selectivity = 0.025 ± 0.102, *P* = 5.104 × 10^−4^) but no bias in the reward epoch (two-sided sign test; mean visual choice-direction selectivity = −0.026 ± 0.154, *P* = 0.211; mean olfactory choice-direction selectivity = −0.021 ± 0.154, *P* = 0.331). To further characterize the ipsilateral bias in the cue epoch, we directly compared the magnitude of selectivity between the ipsilateral-selective and contralateral-selective neurons. In both modalities, the magnitude of the selectivity was not significantly different (two-sided Wilcoxon rank-sum test; *P* = 0.48 for visual choice-direction selectivity, *P* = 0.07 for olfactory choice-direction selectivity). These results show no reliable evidence for biased choice-direction encodings in the PRC, which is in line with recent findings in other cortical areas^7,34^. The choice-direction selectivity was highly consistent across the cue modalities during both epochs (*r* = 0.593, *P* = 5.180 × 10^−21^ for the cue epoch; *r* = 0.858, *P* = 2.837 × 10^−61^ for the reward epoch; Fig. 2c), and the PRC neurons were not tightly clustered as a choice-direction selective subpopulation and the others but rather showed graded selectivity as reported in other cortical areas^7,35^. The neurons which showed significant selectivity across both modalities were sparser in the cue epoch (26% of selective neurons) than in the reward epoch (48% of selective neurons), but we found that the neurons classified as non-selective during the cue epoch (48.79%, 101 of 207 neurons) showed a moderate correlation between the cue modalities (*r* = 0.357, *P* = 2.505 × 10^−4^; inset of Fig. 2c). This suggests that even such non-selective neurons might convey a fraction of the choice-direction information. Altogether, these results suggest that sensory inputs from different modalities evoke similar response patterns across the PRC neurons according to learned cue–target associative relationships.

A potential caveat in this conclusion, however, is that the choice-direction selectivity in the PRC can be attributed to some fundamental behavioral or contextual variables such as body posture, non-orienting movements, and spatial view^30,34,36–39^. To evaluate the possible influence of those variables, we performed two controls. First, we monitored head angles of the animals (*n* = 2) during neural recordings (Supplementary Fig. 2a–c) using a head-mounted accelerometer^40^. To quantify the influence of body posture and view angle on the PRC neural responses (*n* = 105), we compared prediction performance between linear-regression models with the choice and *x*-axis (interaural axis) head angle by computing correlations between the neural responses and the model prediction across trials (Supplementary Fig. 2d). The performance of the choice model was higher than the *x*-axis head angle model in both of the cue and reward epochs by 4.28% ± 10.98% and by 32.07% ± 34.8% (median ± median absolute deviation), respectively. The responses of the majority of the PRC neurons were better explained by the choice than the *x*-axis head angle (two-sided sign test; cue epoch, *P* = 0.019; reward epoch, *P* = 1.365 × 10^−7^). Second, a delay period was inserted between the target choice and reward onsets to dissociate the influence of anticipatory licking behavior^41^ and spatial view (position) from the choice-direction selectivity in the reward epoch. We found that many of the neurons (69.52%, 73 of 105 neurons) encoded the choice directions after the onset of the reward (0–400 ms after reward onset) in accordance with the reward-epoch responses shown in Fig. 2c. The majority of these selective neurons (71.23%, 52 of 73 neurons) showed stronger selectivity after the reward onset than during the reward delay (two-sided sign test; median difference in the magnitude of selectivity = 0.08, *P* = 3.713 × 10^−4^; Supplementary Fig. 2e). Although the other neurons (28.77%; 21 of 73 neurons) showed stronger selectivity during the reward delay than after the reward, the size of such bias (that is, the difference in magnitude between these epochs) was relatively small (0.058 ± 0.061; *n* = 21 neurons) as compared to the bias to the reward onset (0.159 ± 0.122; *n* = 52 neurons). Taken together, these results suggest that fundamental variables such as body posture, non-orienting movements, and spatial view themselves are not major factors for the choice-direction encodings in the PRC.

### Dynamic temporal encoding patterns in the PRC

**Fig. 3:**
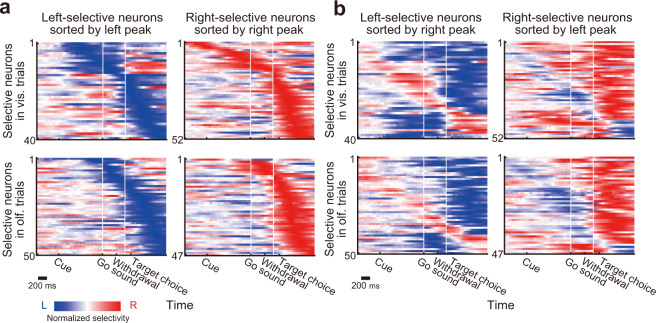
Time-varying choice-direction selectivity in the individual PRC neurons. **a** Temporal response patterns of left-selective and right-selective subpopulations. In each modality, neurons were classified by their peaks of the choice-direction selectivity (Fig. 2a, Supplementary Fig. 1). The neurons were sorted according to the time of their peak selectivity. To visualize the entirety of time-varying selectivity, all the segments with and without statistical significance are shown, and the selectivity of each neuron was normalized to its peak. **b** Same as in **a**, except for that the neurons were sorted by their peak selectivity to the opposite choice direction.

Thus far, we showed that the PRC neurons encoded the choice directions across the different cue modalities and that such signals were apparent at multiple epochs of the task (Fig. 2, Supplementary Fig. 2). Given the reduced correlation of temporal response patterns between the choice directions (Fig. 1d–e), it is possible that the individual neurons flexibly tuned to the different choice directions at different time points of the trial duration. To characterize how each neuron represented the choice directions over the trial duration, we classified the neurons into two groups based on their peak selectivity: left-selective and right-selective neurons. As shown in Fig. 3a, we found similar numbers of selective neurons for both choice directions. As expected, the individual neurons showed selectivity to the opposite choice direction in time points other than their peak responses. Remarkably, when we sorted these neurons by their peak responses to the opposite choice (that is, left-selective neurons by right peaks and right-selective neurons by left peaks), encoding patterns nearly tiling the entire trial duration appeared (Fig. 3b). These results suggested flexible engagements of the individual neurons for the different choice directions rather than the recruitment of distinct subpopulations for each choice direction.

**Fig. 4:**
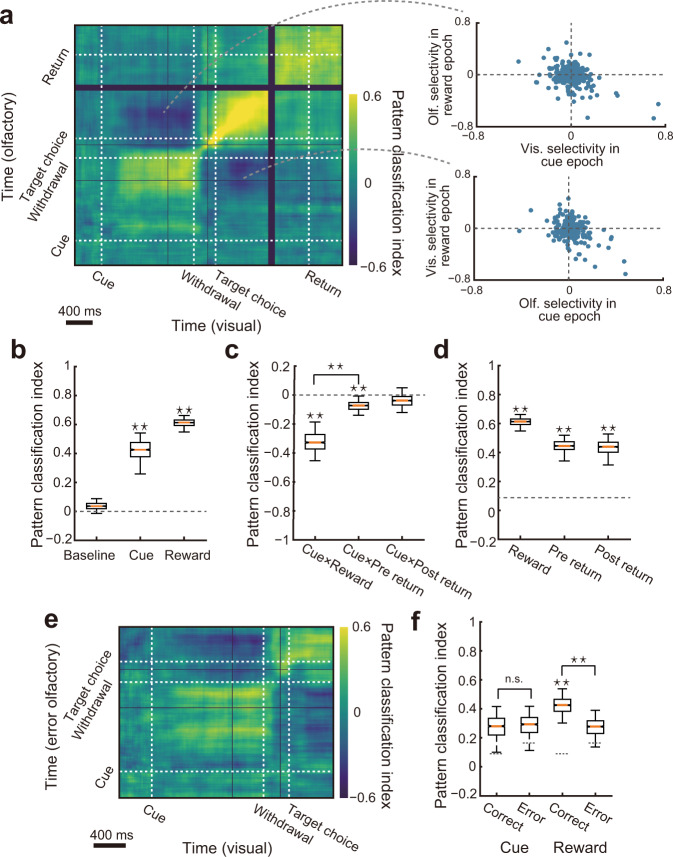
Time-resolved pattern analysis for temporal changes of choice-direction selectivity. **a** Performance of the pattern classification analysis (left). White lines correspond to the onset of cue, withdrawal, target choice, and return movement to the central port (target-choice offset). We built a classifier using neural responses in the correct visual trials to discriminate the choice directions and tested them with neural responses in the correct olfactory trials (*n* = 207 neurons). Temporal inversions of the choice-direction selectivity between the cue and reward epochs are shown by scatter plots (right). **b** Mean classification performance during the baseline, cue, and reward epochs. **c** Mean classification performance across two different epochs. **d** Mean classification performance during the reward, pre-return, and post-return epochs. The dashed line indicates the 97.5th percentile values of the baseline distribution shown in **b**. **e** A classifier tested with the erroneous olfactory trials (*n* = 119 neurons). **f** Mean classification performance in the erroneous trials compared with that in the correct trials. Dashed lines indicate the 97.5th percentile values of the baseline distributions in the correct and erroneous trials. In box plots: orange line, median; box limits, 25th and 75th quartiles; notch limits (1.57 × interquartile range)/√*n*; whiskers, 95th percentile range (two-sided) of the distribution. Asterisks indicate statistical significance based on estimated *P* values (*P* < 0.05; Methods), and n.s. indicates insignificance.

We next sought to understand how such dynamic encoding patterns in the PRC appear and evolve through the trial duration as a population. A time-resolved pattern analysis^42^ (“Methods”) was performed to visualize the temporal evolution of the choice-direction encoding patterns across the PRC neurons (*n* = 207). Given the overall correlation of the choice-direction selectivity between the cue modalities during the epochs where such encodings peaked (Fig. 2b–c), we focused on modality-independent encoding patterns (for comparison between the visual and olfactory trials, see Supplementary Fig. 3). The population response pattern evolved with two time-stable states (Fig. 4a). An encoding pattern was sustained during the presentation of the cue and was followed by a transient pattern during the movements towards the target ports. Soon after the rats chose a target port, the encoding pattern settled again into a stable state. To test for reliability of such encodings, we computed the mean performance of the classifier during the cue and reward epochs and compared them with a baseline epoch (−400 to 0 ms before the cue onset). As shown in Fig. 4b, the choice directions were decoded during both epochs above chance (cue epoch: *P* ≈ 0.002; reward epoch: *P* < 0.001). Importantly, the encoding patterns were substantially inverted between the cue and reward epochs (scatter plots in Fig. 4a). The mean classification performance across those epochs revealed reliably inverted encoding patterns (*P* < 0.001; Fig. 4c). It is noteworthy that the inverted state was time-stable (Fig. 4a). This indicates that the time-varying choice-direction encodings which we found in the previous section (Fig. 3b) are explicitly aligned to between the cue and reward epochs. In other words, the inverted encodings might be a form of coherent representation across different epochs, which is mediated by the individual PRC neurons.

However, it is still possible that the inverted encoding patterns were due to the reversal of orienting movements between these epochs. To directly test this, we analyzed neural responses around the time when the animals left the target ports in preparation to the next trials (Supplementary Fig. 4): pre-return (−400 to 0 ms before the choice offset) and post-return epochs (0 to 400 ms after the choice offset). These epochs did not show the equivalent level of inverted patterns from the cue epoch (cue × pre-return epochs: *P* ≈ 0.016; cue × post-return epochs: *P* ≈ 0.1858; comparison between cue × reward epochs and cue × pre-return epochs: *P* ≈ 0.023; Fig. 4c) despite the fact that the choice-direction information was robustly represented during both pre-return and post-return epochs (*P* < 0.001; Fig. 4d). These results indicated little influence of orienting movements on the dynamic choice-direction encoding patterns.

We also asked whether the behavioral performance affect the neural responses by performing the pattern classification analysis with correct visual and erroneous olfactory trials (*n* = 119 neurons with a sufficient number of erroneous trials; Fig. 4e). As shown in Fig. 4f, the mean classification performance during both epochs was the chance level in the erroneous trials (error cue: *P* ≈ 0.1389; error reward: *P* ≈ 0.1349). For an accurate comparison, the classification performance for correct trials was obtained from the visual and downsampled olfactory trials (“Methods”). After the downsampling, the mean classification performance was still above chance for the reward epoch but did not reach significance in the cue epoch (correct cue: *P* ≈ 0.073; correct reward: *P* < 0.001), suggesting higher trial-by-trial variability in the cue epoch. We tested whether the classification performance during each epoch decreased from the correct trials to erroneous trials and found a significant reduction only in the reward epoch (cue: *P* ≈ 0.5385; reward: *P* < 0.001). These different results suggest distinct neural computations underlying the choice-direction encodings in these epochs.

### Population structure and single-neuron representations

**Fig. 5:**
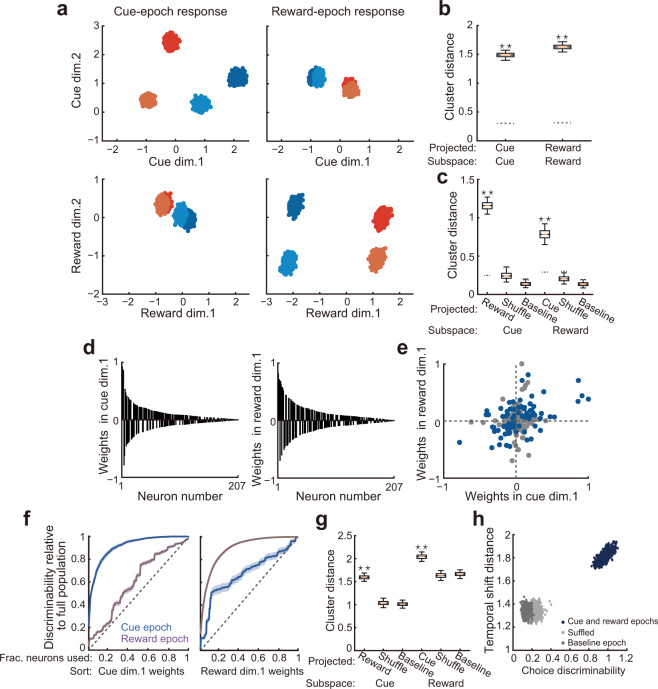
Principal component analysis of neural population responses. Population responses during the cue and reward epochs projected onto the first two dimensions of cue-epoch and reward-epoch subspaces. Blue, left target choice in visual trials; pale blue, left target choice in olfactory trials; red, right target choice in visual trials; orange, right target choice in olfactory trials. Each point corresponds to the population response in a subset of trials. **b**, **c** Comparisons between the across-condition distances and within-condition distances. Dashed lines indicate the 97.5th percentile ranges of the within-condition distances. **b** Distances obtained from projections onto the corresponding subspaces. **c** Distances obtained from projections onto the interchanged subspaces. **d** Weights of the individual neurons in the first dimensions of the cue-epoch (left) and reward-epoch (right) subspaces shown in **a**. The values were normalized by the magnitude of the highest weighting value and were ordered by the magnitude. **e** Correlation between the neural weights in the first dimensions of the cue and reward subspaces shown in **d**. Blue points, neurons with significant choice-direction selectivity (*P* < 0.05) across the cue and reward epochs. **f** Choice-direction discriminability by neural populations with increasing numbers of neurons. Neurons were arranged by the weighting values on the first dimension of the cue epoch (left) or the reward epoch (right). Values were normalized to the discriminability by the full population. Lines and shaded areas represent mean and 95th percentile range, respectively. Dotted lines indicate the increase expected by random incorporation of the neurons. **g** Temporal shifts in representational geometry. The same shuffled data as in **c** was shown here. We considered the baseline-epoch responses to be a chance level. **h** Relationships between the temporal coherence and dynamics of the PRC population responses. Each single point corresponds to the population response in a subset of trials. The discriminability by the cue-epoch and reward-epoch responses is shown as blue points. The same for the baseline-epoch responses and shuffled responses are shown as deep gray and light gray points, respectively. In box plots, the same convention as in Fig. 4.

To investigate how such coherent response patterns appeared and worked under the entire population structure, we identified neural subspaces for the cue and reward epochs by performing the principal component analysis (PCA). Since our focus was to compare population response patterns between these different epochs, PCA was applied to time-averaged cue-epoch and reward-epoch responses across the conditions (that is, PCA on neurons × conditions matrix for each epoch)^43,44^. This allowed us to clarify the population-encoding structure derived from the cue-epoch and reward-epoch responses and directly investigate the interrelationship between them. We projected the cue-epoch and reward-epoch responses onto the first two dimensions of the cue-epoch and reward-epoch subspaces (Fig. 5a, Supplementary Fig. 5a). The different choices and cue modalities were separated when we projected the neural responses onto the corresponding neural subspaces (Fig. 5a, cue epoch in upper left; reward epoch in bottom right). Although we observed highly correlated choice-direction selectivity between the visual and olfactory trials (Fig. 2c), the modality information was evident in these plots. In both epochs, the first and second dimensions mainly captured the difference of the choice directions and the cue modalities, respectively. We quantified separation among the four different conditions in those plots by comparing the average distance among responses under the different conditions (across-condition distance) with the average distance among responses under the same conditions (within-condition distance). The former distance indicates the discriminability of the different conditions, and the latter indicates the variability of the population responses within each condition. The results revealed that the across-condition distance was significantly larger than the within-condition distance in both projections, indicating reliable encodings of the different conditions during both epochs (*P* < 0.001; Fig. 5b and “Methods”). We also determined whether these neural subspaces depended on the entire neural population or only a fraction of the neurons. We found that neural weights were highly distributed across the neurons (Fig. 5d, Supplementary Fig. 5b) with no neurons showing zero weight. This suggests that these subspaces reliably reflect coordinated response patterns across the PRC neurons. To directly investigate the relationship between the cue-epoch and reward-epoch responses, we projected the population responses onto the interchanged neural subspaces (that is, the cue-epoch responses onto the reward-epoch subspace and the reward-epoch responses onto the cue-epoch subspace)^4,8^. The results showed that the choice directions but not the cue modalities were separable through the epochs (Fig. 5a, cue-epoch responses onto reward-epoch subspace in bottom left; reward-epoch responses onto cue-epoch subspace in upper right). To test the reliability of the choice-direction discriminability sustained across the epochs, we compared the across-condition distance for different choice directions on the first dimension with the within-condition distance. As shown in Fig. 5c, in both subspaces, we found cluster separations above chance (reward-epoch responses in cue-epoch subspace: *P* < 0.001; cue-epoch responses in reward-epoch subspace: *P* < 0.001), revealing that the choice-direction information was reliably sustained between those epochs. We also projected the neural responses during the baseline epoch onto the same neural subspaces as negative controls (Supplementary Fig. 5c). This analysis revealed no significant cluster separations (*P* ≈ 0.998 for cue-epoch subspace; *P* ≈ 1 for reward-epoch subspace; Fig. 5c), indicating no reliable encodings of the choice-direction information. We then tested whether the temporal response patterns of the individual neurons contributed to the observed discriminability by projecting randomly shuffled data where the temporal response patterns of the individual neurons were collapsed (Supplementary Fig. 5d). The results showed no significant separations (*P* ≈ 0.998 for shuffled reward-epoch responses in cue-epoch subspace; *P* ≈ 0.999 for shuffled cue-epoch responses in reward-epoch subspace; Fig. 5c), suggesting that the coherence between the cue and reward epochs at the single-neuron level was critical for the discriminability observed at the population level.

It might be possible that only a handful of neurons had strongly contributed to both cue-epoch and reward-epoch subspaces, thus resulting in the shared neural dimensions (that is, the first dimensions which consistently discriminated the choice directions) shown in Fig. 5a. However, this was not the case. As shown in Fig. 5e, the values of neural weights in the first dimensions of the cue-epoch and reward-epoch subspaces showed neither explicit clusters nor a tight correlation but rather exhibited a continuous distribution with a moderate correlation (*r* = 0.337, *P* = 7.12 × 10^−7^). This suggests that many neurons dynamically changed their contributions to the shared dimensions across the epochs, while a degree of temporal coherence supporting those shared dimensions was maintained at the population level. Diverse relationships of the neural weights were observed even among the neurons with significant choice-direction selectivity (computed using ROC analysis in Fig. 2c) across the epochs (blue points in Fig. 5e). This result indicated heterogeneity of the temporally coherent single-neuron responses in their contributions to the entire population structure. In other words, each of those neurons might play a unique role in population encodings under different computations.

To further establish population-level encoding^7,9,35,45^ in the PRC, we directly examined the distribution of the choice-direction information over the individual neurons. We asked what fraction of the neurons is necessary to achieve the choice-direction discriminability (that is, the across-condition distance for different choice directions on the first dimension of a given epoch) which is equivalent to the entire population (“Methods”). The number of neurons incorporated was gradually increased, beginning with ones with the highest magnitude of the weighting values on the first dimension of the cue or reward epoch (Fig. 5f). As expected from the shape of the distribution in Fig. 5d, when the neurons were sorted by the cue-epoch weights, the choice-direction discriminability in the cue subspace steeply increased with the incorporation of neurons (left in Fig. 5f). However, the top 73.43% of the neurons were necessary to achieve the discriminability as high as the entire population. Similarly, when the neurons were sorted by the reward-epoch weights, the discriminability in the reward subspace reached as high as the full population after including 85.02% of the neurons (right in Fig. 5f). These results support population-level representations in which information is distributed across many individual neurons. On the other hand, the choice-direction discriminability more gradually increased for the epochs to which the orders of neurons were not aligned (Fig. 5f), highlighting the dynamic changes in the neural weights between the epochs observed in Fig. 5e. We found that nearly all the neurons were necessary to achieve the discriminability as high as the full population (neurons sorted by cue-epoch weights, 100%; neurons sorted by reward-epoch weights, 97.58%), indicating that the choice-direction representations across the two epochs were distributed widely over the entire population. However, the discriminability in the epochs in which the neurons were indirectly sorted more steeply increased than the linear increase which is expected when the neurons were added in a random order (two-sided Kolmogorov–Smirnov test; cue epoch, *P* = 5.721 × 10^−15^; reward epoch *P* = 2.078 × 10^−5^). As shown in Fig. 5f, by incorporating a small subset of the neurons (~three neurons), those curves deviated from the linear increase, and the deviation was sustained through almost all the parts of the curves. Therefore, the choice-direction information in the cue and reward epochs was highly distributed across the PRC neurons, but its coherence was reliably sustained at the population level.

### Efficient encoding of choice directions and task epochs

As shown in Fig. 5a, the population response patterns representing the choice directions were substantially inverted on the first dimension when we compared the cue-epoch and reward-epoch responses in the same neural subspace (comparison between horizontally arranged plots in Fig. 5a). In contrast, when the population responses from one of these epochs were projected onto the cue-epoch and reward-epoch subspaces (comparison between vertically arranged plots in Fig. 5a), the relative positions of the different choice directions were preserved, indicating that the directions (that is, signs) of the first dimensions were consistent. Therefore, these results suggested that information of the different epochs was also represented in these shared first dimensions by flexible changes in the representational geometry. The inverted geometry was consistent with the dominant temporal response pattern observed in Fig. 4a. To test the reliability of the temporal inversions, in the first dimensions of each subspace, we computed the average distance between population responses under the same conditions in the different epochs (for example, distance from the cue-epoch responses to reward-epoch responses in the cue subspace). We compared those distances with the average distance from one of those epochs to the baseline epoch (for example, distance from the cue-epoch responses to baseline-epoch responses in the cue subspace), which is equivalent to the range of chance-level cluster shifts caused by the absence of the choice-direction encodings (that is, overlapped clusters in Supplementary Fig. 5c). As shown in Fig. 5g, the results showed larger task-epoch dependent shifts of the representational patterns than expected by chance (*P* < 0.001 for cue-epoch subspace; *P* < 0.001 for reward-epoch subspace). The discriminability was diminished when the temporal patterns of individual neural responses were collapsed by data shuffling (*P* ≈ 0.986 for shuffled reward-epoch responses in cue-epoch subspace; *P* ≈ 0.999 for shuffled cue-epoch responses in reward-epoch subspace). These results suggested that the coherent response patterns at the individual neurons mediated flexible changes for the task-epoch representations. Finally, we asked how these flexible encodings for the different epochs (Fig. 5g) were related to the coherent choice-direction encodings across those epochs (the across-condition distance in Fig. 5c). We compared the variability of the choice-direction discriminability and the temporal shift distance across subsets of trials (Fig. 5h) (“Methods”). We found that transition of the population response between the cue and reward epochs resulted in a high correlation between these two values (*r* = 0.719, *P* = 1.369 × 10^−159^), suggesting that the PRC population specifically tuned to support both coherence and dynamics of neural representations between these epochs. Such a strong correlation was absent in the transition of population response between the baseline epoch and the cue or reward epochs (*r* = 0.021, *P* = 0.501). Importantly, the correlation depended on the temporal response patterns in the individual neurons (*r* = 0.088, *P* = 0.005 for shuffled responses), suggesting that the coherent single-neuron responses were critical to efficiently enhance the coherence and dynamics of the neural representations. Taken together, these findings indicated that the PRC population reconciles the coherent representations of choice directions with the dynamic representations for different epochs via temporally flexible but structured neural responses (i.e., inverted selectivity between epochs).

## Discussion

We investigated how coherent single-neuron representations across different epochs can be reconciled with the temporal dynamics of population structure. Individual neurons in the PRC modulated their firings according to the choice directions in each trial, and many neurons inverted such selectivity between the cue and reward epochs. Despite the structured temporal response patterns, we found a dynamic reorganization of the population-encoding structure between the epochs (that is, neural subspaces supported by different coordination patterns across neurons). Yet, we also found shared neural dimensions between the epochs, where the choice-direction information was consistently represented. Those dimensions depended on the coherent temporal patterns of the single-neuron responses. These findings indicated that the temporally coherent single-neuron responses contribute to the dynamic population structure.

It is recently suggested that choice-related encodings observed in higher-order cortical areas are not necessarily abstract signals but rather parsimoniously explained by combinations of fundamental behavioral and contextual variables such as spatial position and head angle^37^. Additionally, a recent study in rats revealed that neurons in the lateral entorhinal cortex are sensitive to subtle changes of egocentric view^38^. In contrast to these findings, our results revealed that the choice directions were a better predictor for the PRC neural responses than other major variables previously noted, namely, body posture, non-orienting movements, and spatial view (Supplementary Fig. 2). Although those individual factors could influence the PRC responses, they were not unique to evoke the PRC responses. Thus, we conclude that the choice-direction encodings in the PRC are an abstract signal integrating various computations related to the choice at each epoch.

How does the nature of the choice-direction encodings differ between the cue and reward epochs? In the cue epoch, we did not find a significant difference between the choice-direction encodings in the correct and erroneous trials (Fig. 4e–f). Given that those neural responses are better explained by the choice directions than by the head angles (Supplementary Fig. 2d), the cue-epoch responses might reflect internal signals such as decision or motor preparation driven by reward expectation^16,46–49^. Contrary, in the reward epoch, the choice-direction encodings significantly decreased in the erroneous trials, conveying the reward signals in conjunction with the choice directions (Supplementary Fig. 2d–e). Those distinct choice-related representations were not expected by previous studies, which highlight perceptual and mnemonic representations in the PRC^19–22,50–53^. However, a recent study suggests that the PRC is also involved in value-based decision making^54^. In light of widespread anatomical connections^23–25^, this region might carry diverse information besides perceptual and mnemonic signals depending on neural computations required for the task^55^.

The above results emphasize the different neural computations underlying the choice-direction selectivity. In spite of the explicit difference, the PRC neurons often showed the choice-direction encodings in both epochs, suggesting that they support associative representations of choice and its outcome. This agrees with a traditional view, which holds that the PRC supports associative memory^16,18–22^. Several studies showed that temporally sustained responses were prevalent in the PRC^16,26,27^. Such sustained responses were found in studies focusing cue and memory epochs and were considered to serve as stable representations of targeted information across time epochs, which enable typical functions of the PRC including recognition memory^56^. Our finding of the inverted response patterns was not predicted by those previous studies but suggested its advantage in supporting both temporal coherence and flexibility of neural representations. Moreover, an important finding in our study is that the discriminability of the choice directions and task epochs was highly correlated (Fig. 5h). This encoding strategy provides a synthesized view upon the roles of the PRC in forming integrated representations of information with shared features and in discriminating between them. Although those functions have been generally tested in visual perception, our findings emphasize its multimodal nature^54^.

It is worth mentioning that the neural weights of individual neurons varied between the cue and reward epochs. In spite of the heterogeneity in individual neurons, at the population level, the neural weights were moderately correlated between the epochs, resulting in the coherent choice-direction representations (Fig. 5e–f). Heterogeneous response properties across neurons are often observed in high-order cortical areas^29,31,39,57–59^ and are considered to increase the number of dimensions that can be represented by a neural population^8,60–63^. The moderately correlated neural subspaces between the cue and reward epochs might enable integrated processing of a targeted variable and contextual information as reported by previous studies^26,27^. Although we did not detect any explicit clusters based on the response property of the PRC neurons (Fig. 5e), it is still possible that they can be categorized into subpopulations based on different projection or cell types^13^. According to this idea, the heterogeneity among the PRC neurons possibly reflects different information routing to downstream regions such as the lateral entorhinal cortex and the hippocampus. For instance, the neurons strongly inverted the choice-direction encodings between the cue and reward epochs can contribute to pattern separation served by the CA3 region^64^. Also, the neurons carried more choice-direction information in the reward epoch might contribute to integrated reward representations in the CA1 region, in which individual neurons convey multiple reward positions^65^. Given the potentially various anatomical properties of individual neurons, future studies should combine analytical and rigorous anatomical approaches to gain a deeper understanding of how a given population contributes to a larger process carried across multiple brain regions^13^.

## Methods

### Subjects

Seven male Long-Evans rats (Shimizu Laboratory Supplies, Kyoto, Japan) weighting 278–375 g at the beginning of the training were individually housed and maintained on a laboratory light/dark cycle (lights on 8:00 A.M. to 9:00 P.M.). Rats were placed on water restriction with *ad libitum* access to food. The animals were maintained at 80% of their baseline weight throughout the experiments. All experiments were implemented in accordance with the guidelines for the care and use of laboratory animals provided by the Animal Research Committee of the Doshisha University with its approval.

### Behavioral apparatus

The behavioral apparatus (Fig. 1a) has been previously described^13,66^. An operant chamber (O’Hara, Tokyo, Japan) with three ports in the front wall for nose-poke responses was enclosed in a soundproof box (Brain Science Idea, Osaka, Japan). Each port was equipped with an infrared sensor to detect the animals’ nose-poke responses. Visual cues were presented using white light-emitting diodes (LEDs) (4000 mcd; RS Components, Yokohama, Japan) placed on the left and right walls of the operant chamber, as shown in Fig. 1a. Cue odors were presented via the central port through a stainless tube. The odors were mixed with pure air to produce a 1:10 dilution at a flow rate of 60 ml/min using a custom-built olfactometer (AALBORG, Orangeburg, NY). Water rewards were delivered from gravity-fed reservoirs regulated by solenoid valves (The Lee Company, Westbrook, CT) through stainless tubes placed inside of the left and right target ports. We controlled stimulus and reward deliveries and measured behavioral responses using Bpod and Pulse Pal^67^ (Sanworks, Stony Brook, NY).

### Two-alternative forced-choice task

Each trial started when the rats poked their snout into the central port (Fig. 1b). After a variable delay (200–600 ms, uniform distribution), a cue randomly selected from four sensory stimuli (left/right LED for visual modality, S(+)/R(−)-2-octanol for olfactory modality) was delivered. If the rats successfully maintained their nose in the central port during 1 s after the cue onset, the “go” sound was delivered, and they were allowed to withdraw from the central port and to choose either left or right target port based on the task rule (Fig. 1a). The presentations of the cue and the go sound were terminated by the withdrawal from the central port. When the rats left the central port without waiting for the go sound, the trial was canceled and followed by a 5 s punish intertrial-interval. Only correct choices were immediately rewarded by a drop of water (13 µl for five rats and 16 µl for two rats) from the target port. Rats performed 861 ± 232 trials in a daily recording session (seven rats, 50 sessions).

For two of seven rats (Supplementary Fig. 2), a 500-ms delay period was inserted between the onset of the target choice and the reward onset to assess the potential influence of non-orienting movements (licking) and spatial view on neural responses.

### Training

We trained the rats step-by-step to perform the task described above. The training period typically lasted 4–8 weeks. First, the rats were trained to poke into the central port and then collect the water reward (20 µl) from the left or right target port. We gradually extended the duration of the central poke by delaying the go sound up to 1 s after the poke onset. Next, the rats were trained to discriminate the odor cues based on the same contingencies as the recording sessions. A variable delay (200–600 ms) was inserted before the cue onset. After the rats became able to successfully discriminate the odor cues (>80%), they were also trained to discriminate the visual cues based on the same contingencies as the recording sessions (>80%). Finally, we interleaved the visual and olfactory trials within a session and trained the rats to accurately perform the task according to a training performance criterion (>80%).

For the two rats (Supplementary Fig. 2), a reward-delay period was introduced after they acquired the odor discrimination. The reward delay was gradually extended from 100 to 500 ms.

### Mixture of odors

The cue odors, S(+)/R(−)-2-octanol, were mixed in a subset of sessions to increase the difficulty of the olfactory discrimination and thereby obtaining a sufficient number of erroneous trials. For instance, we used a 60/40 ratio in a given session, delivering an odor mixture of 60% S(+)-2-octanol and 40% R(−)-2-octanol. We maintained the odor discrimination accuracy constant (>80%) throughout the recording sessions by adjusting the degree of odor mixing before each session.

### Surgery

Rats were anesthetized with 2.5% isoflurane before surgery, and it was maintained throughout surgical procedures. We monitored body temperature, movements, and hind leg reflex and adjusted the depth of the anesthesia as needed. An eye ointment was used to keep the eyes moistened throughout the surgery. Subcutaneous scalp injection of a lidocaine 1% solution provided local anesthesia before the incision. The left temporalis muscle was retracted to expose the skull during the surgery. A craniotomy was performed over the anterior part of the left PRC (AP −3.5 to −3.24 mm, ML 6.6–6.8 mm relative to the bregma, 3.5–4.0 mm below the brain surface), and a custom-designed electrode was vertically implanted using a stereotactic manipulator. A stainless-steel screw was placed over the cerebellum and served as the ground during the recordings. We used the mean response of all the electrodes as a reference. During a week of postsurgical recovery, we gradually lowered the tetrodes to detect unit activities in the PRC. Electrode placement was estimated based on the depth and was histologically confirmed at the end of the experiments.

### Histology

Once the experiments were completed, the rats were deeply anesthetized with sodium pentobarbital and then transcardially perfused with phosphate-buffered saline and 4% paraformaldehyde. The brains were removed and post-fixed in 4% paraformaldehyde, and 100 μm coronal sections of the brains were prepared to confirm the recording sites.

### Electrophysiological recordings

A custom-designed electrode composed of eight tetrodes (tungsten wire, 12.5 µm; California Fine Wire, Grover Beach, CA) was used for the extracellular recordings. The tetrodes individually covered by a polyimide tube (A-M Systems, Sequim, WA) were placed at a 100-µm separation and typically had an impedance of 150–700 kΩ at 1 kHz. The signals were recorded with Open Ephys acquisition board (Open Ephys, Cambridge, MA) at a sampling rate of 30 kHz and bandpass filtered between 0.6 and 6 kHz. The tetrodes were lowered approximately 80 µm after each recording session, and thereby independent populations of neurons were recorded across the sessions.

### Monitoring of body posture during task performance

We used a head-mounted accelerometer (Intan Technologies, Los Angeles, CA) to obtain postural signals from the animals (*n* = 2) during the electrophysiological recordings (Supplementary Fig. 2). The accelerometer signals (*x*-, *y*-, *z*-axis) were recorded at a sampling rate of 30 kHz and then downsampled to 100 Hz. To precisely detect the body posture of the animals, gravity components of the accelerometer signals were estimated by using a low-pass filter with a cut-off frequency of 2 Hz^40^. To reduce the influence from the head angle that each animal typically preferred^40^, for each axis, the above-processed signals were normalized to the mean and standard deviation in the baseline epoch (−400 to 0 ms before the cue onset).

### Spike sorting and screening criteria of units

All analyses were performed using MATLAB (MathWorks, Natick, MA). To detect single-neuron responses, the spikes were manually clustered with MClust (A.D. Redish) for MATLAB. Only neurons met the following criteria were included for further analyses: (1) units with sufficient isolation quality (isolation distance ≥15); (2) units with reliable refractory periods (violations were <1% of all spikes); and (3) units with sufficient mean firing rates in the 1 s after the cue onset (>0.5 Hz). On average, we detected 10.9 ± 7.4 neurons in a single recording session, and 6.2 ± 3.4 neurons survived these quality criteria (total of 312 neurons from seven rats).

### Selective responses to choice directions

To evaluate the selective responses to different directions of choices, we computed a choice-direction selectivity^68^. We first grouped correct trials into four types based on the cue modality (visual or olfactory) and the target choice (left or right). For each modality, we independently computed the choice-direction selectivity by using ROC analysis^69^. The choice-direction selectivity was obtained from the area under the ROC curve (AUC) and defined as 2 × (AUC − 0.5) ranging from −1 to 1. In our analysis, a positive value indicated a neuron selectively fired to the left target choice, and a negative value indicated the opposite. A value of zero indicated the absence of choice-direction selective responses. To determine statistical significance (*P* < 0.05), we used permutation tests (1000 iterations). For visualization of the temporal patterns of the choice-direction selectivity in individual neurons (Figs. 2a and 3, Supplementary Figs. 1 and 2b), mean firing rates were computed in 10 ms time windows (smoothed with a Gaussian, *σ* = 30 ms), and we then computed the choice-direction selectivity at each time point from those data.

### Estimation of *P* value in bootstrapping procedure

We evaluated the statistical significance of the decoding analysis and the state space analysis with a bootstrapping procedure^8^. We estimated the *P* value for the bootstrapping procedure by computing the ratio (1 + *X*)/(*N* + 1), where the number *X* indicates overlapping data points between the two distributions, and the number *N* indicates iterations. Since we used 1000 bootstraps, two distributions with no overlap resulted in *P* < 0.001, and two distributions with *x*% overlap resulted in *P* ≈ *x* /100.

### Decoding analysis

We employed a cross-temporal pattern analysis^42,58^ to investigate the temporal changes in the choice-direction selective responses in the PRC. Neural responses were pooled across the recording sessions to maximize the number of neural responses included in the analysis. Here, we refer to the pooled pseudo-population of the PRC neurons (*n* = 207 from five rats) as the full population. The instantaneous firing rate of each neuron was estimated by the spike counts in a 150-ms sliding window (10 ms increment). We computed the above described choice-direction selectivity from the instantaneous firing rates for each neuron independently for visual and olfactory trials. In this manner, we generated two independent population vectors for the full population (neurons × time matrix each for the cue modalities). We obtained a pattern similarity index by computing the Fisher-transformed Pearson correlation (*r*′) between these two population vectors. This index provided the pattern similarity for both equivalent and different time points (e.g., Fig. 4a). A positive value was interpreted as the evidence for a reliable choice-direction encoding irrespective of the cue modality.

To estimate the mean performance from the pattern classification analysis, we randomly resampled the neurons (the same number of neurons as the neural population analyzed) and computed the choice-direction selectivity in the visual and olfactory trials. Neural responses were aligned to the withdrawal onset from the central port (for the cue epoch), target-choice onset (for the reward epoch), and target-choice offset (for the two return epochs). For the return-epoch responses, we analyzed neural responses before and after the animals left the target ports in preparation for the next trials (pre-return epoch: −400 to 0 ms before leaving a target port: post-return epoch: 0–400 ms after leaving a target port; Supplementary Fig. 4). Only trials where the animals directly returned from the target port to the central port for the next trial were included in the analysis. These data allowed us to compute the pattern similarity indices within an epoch or across two different epochs. To investigate the choice-direction encodings during these epochs, we averaged the performance within each of the epochs (the cue, reward, pre-return, and post-return epochs). To obtain a baseline performance, we averaged the classification performance during the baseline epoch, −400 to 0 ms before the cue onset. We also quantified the pattern similarity of the neural responses between two epochs (for simplicity, we here refer to these epochs as A-epoch and B-epoch) by averaging the following two pattern classifications: pattern classification for A-epoch responses in visual trials and B-epoch responses in olfactory trials, and for A-epoch responses in olfactory trials and B-epoch responses in visual trials (e.g., Fig. 4c). We repeated the above processes 1000 times to obtain a distribution of 1000 different measurements of each pattern classification. To determine the statistical significance, we compared a distribution obtained from 1000 different pattern classification measurements within an epoch with a baseline distribution using the above described estimated *P* value. We considered zero to be a chance level instead of the baseline distribution when we verified the statistical significance of the classification across two different epochs.

### Decoding analysis of erroneous trials

We included 119 neurons recorded in sessions with a sufficient number of erroneous target choices (at least 11 trials for both directions of the target choices) in all the analyses for erroneous trials. Due to the similarity of the choice-direction selectivity between the different cue modalities in correct trials (Figs. 2c and 4a), we computed the pattern similarity indices using the correct visual trials and erroneous olfactory trials to evaluate the influence of erroneous behavioral performance on the choice-direction encodings (Fig. 4e). In the erroneous trials, the animals chose the same target port as the correct trials, but the choices were not made based on the correct cue–target associations. To determine the statistical significance of the pattern classification performance in the correct and erroneous trials, we downsampled the correct olfactory trials to match the number of trials for the erroneous olfactory trials (Fig. 4f). To test whether the classification performance during each epoch decreased from the correct trials to the erroneous trials, we subtracted the classification performance in the erroneous trials from that in the correct trials for each resampling. The distribution of the residual performance was compared with zero by using the estimated *P* value.

### State-space analysis

To understand the population structure and its temporal change between the cue and reward epochs, we performed the PCA. For each of the epochs, we constructed 207 neurons × 4 conditions matrix^43,44^, in which columns contained trial-averaged *z*-scored firing rates of each neuron. The instantaneous firing rate of each neuron (estimated by spike counts in a 150-ms sliding window with 10 ms increment) was converted to a *z*-score by normalizing to the mean and standard deviation of its instantaneous firing rates during the baseline epoch. We then obtained time-averaged firing rates of the neurons each for the cue and reward epochs. By performing PCA on these datasets, we reduced the dimensionality of the PRC population from 207 neurons to two principal components (Supplementary Fig. 5a). We independently performed this analysis for population responses during the cue and reward epochs to obtain the cue-epoch and reward-epoch subspaces.

For data projections onto the above two-dimensional neural subspaces, we randomly selected 25 trials for each of the four trial conditions. *Z*-scored firing rates of each neuron were then obtained by normalizing the data to the mean and standard deviation of its firing rates during the baseline epoch. We averaged the *z*-scored firing rates of each neuron during each of three epochs (the cue, reward, and baseline epochs) to obtain 207 neurons × 4 conditions matrix for each epoch. To visualize the population responses, we projected these data onto the two-dimensional PCA space. This allowed us to obtain a single point reflecting the entire population response for each of the four conditions. We repeated this procedure five times with different subsets of 25 trials, and this allowed us to reduce some degree of the variability among individual trials. To obtain the within-condition distance, we computed the mean of pairwise Euclidean distances for five points in each of the four conditions and then averaged those distances across the conditions to obtain a single value for the within-condition distance. To obtain the across-condition distance, we computed pairwise distances between two sets of five points obtained from two different conditions in the same subspace and then averaged them. This procedure was repeated for all the possible combinations of two different conditions of interest, and we then averaged those distances to obtain a single value for the across-condition distance. We also investigated how the representational geometry for the choice directions changed between different epochs (i.e., the temporal shift of population responses in a neural subspace). This was achieved by computing the mean of pairwise distances between two sets of five points obtained from neural responses under a single condition in two different epochs. The mean distance on the first dimension was obtained for all trial conditions of interest and then averaged to produce a single value for the across-condition distance for the temporal shifts.

To visualize variability across different subsets of trials (Fig. 5a) and test statistical significance, the above analysis was repeated 1000 times with different subsets of resampled trials. The across-condition distances of the projections on the corresponding subspaces (that is, the cue-epoch responses onto the cue-epoch subspace and the reward-epoch responses onto the reward-epoch subspace; Fig. 5a) were compared with a distribution of the within-condition distances using the above described estimated *P* value (Fig. 5b). The across-condition distances larger than the within-condition distance indicated that different trial conditions were reliably discriminated at the population level. For projections onto the interchanged subspaces (that is, the cue-epoch responses onto the reward-epoch subspace and the reward-epoch responses onto the cue-epoch subspace; Fig. 5a), the across-condition distances for the different choice directions on the first dimension were computed in each modality and then averaged. We compared those distances with a distribution of the within-condition distances on the first dimension by using the estimated *P* value (Fig. 5c). Similarly, we projected the baseline-epoch responses as negative controls (Fig. 5c, Supplementary Fig. 5c). Also, to investigate the importance of the temporal response patterns of individual neurons, we projected randomly shuffled data (Fig. 5c, g), where the correspondence between the cue-epoch responses and reward-epoch responses were shuffled among the neurons (Supplementary Fig. 5d). We considered the across-condition distances of the baseline-epoch responses to be chance when we evaluated the temporal shifts of representational geometry between the cue and reward epoch in a neural subspace (Fig. 5g). To understand the relationship between the coherent choice-direction discriminability and the temporal shifts of representational geometry between the cue and reward epochs (Fig. 5h), we averaged the across-condition distances for the different choice directions computed in each of the cue and reward subspaces (Fig. 5c) and summarized the temporal shifts of the representational geometry by averaging the temporal shift distances computed in each of the cue and reward subspaces (Fig. 5g). We directly compared these summarized distances obtained from each condition in Fig. 5h.

To directly evaluate the contributions of individual neurons to the choice-direction encodings (Fig. 5f), we performed the following analysis. We first sorted all the neurons from ones with the highest magnitude of the weighting values on the first dimension of the cue or reward epoch. For these differently sorted populations, we performed cluster projections onto the corresponding subspaces with increasing numbers of neurons. For precise evaluation of each neuron’s contribution, rather than performing PCA on each of differently sized populations independently, we used the same projection data in Fig. 5a (that is, the full population data) and replaced the weighting values of excluded neurons with zero. In each size of the population, the across-condition distance for the different choice directions on the first dimension was computed for each modality and then averaged. Those distances were then normalized to the distance obtained from the full population. We compared the distances from differently sized populations with those from the full population by the estimated *P* value. When the estimated *P* exceeded 0.05, we considered that the number of neurons was sufficient to achieve the discriminability of the choice directions equivalent to the full population.

### Statistics and reproducibility

For reproducibility, behavioral and neural data were obtained from the seven rats. More than 20 well-isolated units were recorded in each of the rats. We excluded data from rats with an insufficient total number of recorded units (*n* < 10 for each). Individual statistical tests are described where referenced in the manuscript. All the data are presented as mean ± standard deviation unless otherwise stated.

### Reporting summary

Further information on research design is available in the Nature Research Reporting Summary linked to this article.

## Supplementary information

Reporting Summary

Supplementary Information

## Data Availability

The datasets analyzed and produced in the present study are available in figshare repository (10.6084/m9.figshare.12611666.v2)^70^.

## Code availability

The custom code used for analyses in the present study is available from the corresponding authors upon reasonable request.
