# Supplementary material for: Dynamic coordination of the perirhinal cortical neurons supports coherent representations between task epochs
Source: Commun Biol. 2020 Jul 30;3:406. doi: 10.1038/s42003-020-01129-3 (PMC7393175; doi:10.1038/s42003-020-01129-3)
Supplement: Supplementary file 1 — Reporting Summary [file 42003_2020_1129_MOESM1_ESM.pdf]

nature research

Corresponding author(s): Tomoya Ohnuki & Junya Hirokawa

Last updated by author(s): Jun 14, 2020

Reporting Summary

Nature Research wishes to improve the reproducibility of the work that we publish. This form provides structure for consistency and transparency in reporting. For further information on Nature Research policies, see Authors & Referees and the Editorial Policy Checklist.

Statistics

For all statistical analyses, confirm that the following items are present in the figure legend, table legend, main text, or Methods section.

n/a	Confirmed	
☐	☒	The exact sample size (*n*) for each experimental group/condition, given as a discrete number and unit of measurement
☐	☒	A statement on whether measurements were taken from distinct samples or whether the same sample was measured repeatedly
☐	☒	The statistical test(s) used AND whether they are one- or two-sided<br>*Only common tests should be described solely by name; describe more complex techniques in the Methods section.*
☐	☒	A description of all covariates tested
☒	☐	A description of any assumptions or corrections, such as tests of normality and adjustment for multiple comparisons
☐	☒	A full description of the statistical parameters including central tendency (e.g. means) or other basic estimates (e.g. regression coefficient) AND variation (e.g. standard deviation) or associated estimates of uncertainty (e.g. confidence intervals)
☐	☒	For null hypothesis testing, the test statistic (e.g. F, t, r) with confidence intervals, effect sizes, degrees of freedom and P value noted<br>*Give P values as exact values whenever suitable.*
☒	☐	For Bayesian analysis, information on the choice of priors and Markov chain Monte Carlo settings
☒	☐	For hierarchical and complex designs, identification of the appropriate level for tests and full reporting of outcomes
☒	☐	Estimates of effect sizes (e.g. Cohen's d, Pearson's r), indicating how they were calculated

Our web collection on statistics for biologists contains articles on many of the points above.

Software and code

Policy information about availability of computer code

Data collection	The data were collected by using open-source devices. Bpod and PulsePal (Sanworks LLC) were used for collecting the behavioral data, and OpenEphys was used for acquiring the neural data.
Data analysis	All data were analyzed using the custom code in MATLAB (ver. 2015a, MathWorks).

For manuscripts utilizing custom algorithms or software that are central to the research but not yet described in published literature, software must be made available to editors/reviewers. We strongly encourage code deposition in a community repository (e.g. GitHub). See the Nature Research guidelines for submitting code & software for further information.

Data

Policy information about availability of data

All manuscripts must include a data availability statement. This statement should provide the following information, where applicable:

- Accession codes, unique identifiers, or web links for publicly available datasets
- A list of figures that have associated raw data
- A description of any restrictions on data availability

The data that support the findings in this study will be available from the corresponding authors upon reasonable request.

Field-specific reporting

Please select the one below that is the best fit for your research. If you are not sure, read the appropriate sections before making your selection.

☒ Life sciences ☐ Behavioural & social sciences ☐ Ecological, evolutionary & environmental sciences

Life sciences study design

All studies must disclose on these points even when the disclosure is negative.

Sample size	No sample-size calculation was performed in this study. The sample size of the behavioral and neural data analyzed in the present study were larger than recent neurophysiological studies in rodent perirhinal cortex (e.g. Ahn & Lee, 2017, 2015) . Also, the sample size of the neural data was comparable to that in previous studies investigating population-level encoding mechanisms (e.g. Elsayed et al., 2016; Rapposo et al., 2014). More than 20 well-isolated units were recorded in each of the seven animals used for the analyses. Data from the four animals were used for analyses of the erroneous trials.
Data exclusions	We only included the data from the animals that well-performed the behavioral task after the training procedures (> 80% accuracy). Also, only the data from the recording sessions, where well-isolated units (units with ID greater than or equal to 15) were detected in the perirhinal cortex, were analyzed. We excluded data from the five animals fulfilling the above criteria because an insufficient number of units (n < 10) were recorded from each of them.
Replication	Experiments were carried out with seven animals. We obtained similar results for the behavioral performance and neural activities among those animals.
Randomization	Not applicable, since we do not have different experimental groups.
Blinding	No blinding was necessary for the experiments.

Reporting for specific materials, systems and methods

We require information from authors about some types of materials, experimental systems and methods used in many studies. Here, indicate whether each material, system or method listed is relevant to your study. If you are not sure if a list item applies to your research, read the appropriate section before selecting a response.

Materials & experimental systems

n/a	Involved in the study	
☒	☐	Antibodies
☒	☐	Eukaryotic cell lines
☒	☐	Palaeontology
☐	☒	Animals and other organisms
☒	☐	Human research participants
☒	☐	Clinical data

Methods

n/a	Involved in the study	
☒	☐	ChIP-seq
☒	☐	Flow cytometry
☒	☐	MRI-based neuroimaging

Animals and other organisms

Policy information about studies involving animals; ARRIVE guidelines recommended for reporting animal research

Laboratory animals	Male Long-Evans rats (278–375 g) were used for this study.
Wild animals	This study did not involve wild animals.
Field-collected samples	This study did not involve samples collected from the fields.
Ethics oversight	All experiments were implemented in accordance with the guidelines for the care and use of laboratory animals provided by Doshisha University with the approval of the Animal Research Committee of Doshisha University.

Note that full information on the approval of the study protocol must also be provided in the manuscript.
